# Sociodemographic Factors Related to Perceived Physical Activity on Chilean Adults after COVID-19 Pandemic

**DOI:** 10.3390/sports12090238

**Published:** 2024-08-30

**Authors:** Rodrigo Gallardo-Rodríguez, Felipe Poblete-Valderrama, Viviana Rodas-Kürten, João Paulo Vilas-Boas

**Affiliations:** 1 School of Education, Universidad Viña del Mar, Viña del Mar 2572007, Chile; 2 Centre of Research, Education, Innovation and Intervention in Sport (CIFI2D), Faculty of Sport, University of Porto, 4200-450 Porto, Portugal; jpvb@fade.up.pt; 3 Department of Sport Science and Physical Conditioning, Universidad Católica de la Santísima Concepción, Concepción 4090541, Chile; fpoblete@ucsc.cl; 4 School of Nursing, Universidad Santo Tomás, Sede Valdivia, Valdivia 5110547, Chile; clararodasku@santotomas.cl; 5 Porto Biomechanics Laboratory (LABIOMEP-UP), University of Porto, 4200-450 Porto, Portugal

**Keywords:** physical activity, sedentarism, COVID-19, sociodemographic

## Abstract

The current study aimed to examine the relationship between sociodemographic variables (i.e., sex, age, marital status, educational level, socioeconomic status, and working mode) and physical activity levels declared by Chilean adults. The sample comprised 483 Chilean adults, 159 men (32.9%) and 324 women (67.1%) aged from 18 to 69 years old (36.5 ± 12.0). The participants completed an ad hoc sociodemographic online survey between December 2022 and March 2023 that included questions about characteristics of participants such as sex, age, educational level, household income, marital status, and working mode. Vigorous, moderate, and walking activities were measured using the International Physical Activity Questionnaire-Short Form (IPAQ-SF), a self-administered questionnaire. Men declared significantly higher vigorous and moderated physical activity than women. People aged 18 to 25, single or unmarried, and with the lowest household income, showed significantly higher scores in vigorous physical activity than those aged 26 to 45, cohabiting with a partner or married, and middle household income, respectively. Regarding working mode, people working at their job site said walking more than people not working, working in a hybrid mode, and working online. Our findings suggest that promoting strategies that increase physical activity during the pandemic is necessary to avoid health problems.

## 1. Introduction

Research into the influence of the coronavirus disease 2019 (henceforth COVID-19) pandemic on physical activity has been subject to growing attention worldwide [1,2,3]. The conditions of high or low participation in physical exercise activities vary depending on the context, and the COVID-19 pandemic has significantly decreased physical activity levels [4]. Sociodemographic factors such as sex, age, socioeconomic status, educational level, and marital status have also been consistently associated with the level and type of physical activity [5,6,7]. However, the people’s working mode is another aspect to consider in this field of study, given the effects that working in the workplace, working remotely, and non-working during the COVID-19 pandemic lockdown have had on higher or lower levels of physical activity [8]. 

### 1.1. Association between Chileans’ COVID-19 Pandemic Context and Physical Activity

The first case of COVID-19 in Chile was in March 2020, marking the beginning of a unique and challenging period. Since then, several public health measures have been implemented, including lockdowns, quarantines, confinement, and reduction of maximum capacity in public places. In August 2020, Chile ranked first in Latin America in the number of PCR and antigen tests and was also the first country in the zone to start the mass vaccination process with the fourth dose against this disease [9].

The COVID-19 pandemic has impacted people’s health habits despite the abovementioned sanitary actions. Scientific evidence suggests that COVID-19 confinement has influenced physical activity, in which people declared that they had experienced a decrease in physical activity and an increasingly sedentary lifestyle [10]. Promoting physical activity in Chile during the COVID-19 pandemic could protect against health problems, which are consequences of physical inactivity at the national level [11,12].

In this context, the quality of life of Chilean adults during the COVID-19 confinement has been influenced by different variables, including the level of a sedentary lifestyle and physical activity. Being physically inactive increases the probability of having a lower quality of life during the pandemic lockdown [13]. The relationship between better quality of life and physical activity could be because it is well known that persons performing regular physical activity perceive themselves as having more autonomy, personal growth, purpose in life, and self-acceptance [14]. In addition, physical activity improves physical and mental health. The WHO recommends that adults perform 150–300 min of moderate exercise or 75–150 min of vigorous exercise weekly, as it is a protective factor against metabolic, cardiac, anxious, and depressive problems and contributes to increased life expectancy [15]. Nevertheless, some sociodemographic factors could be determinants of physical activity among adults during the COVID-19 pandemic.

### 1.2. Influence of Sociodemographic Factors on Physical Activity

Among demographic variables related to physical activity levels, socioeconomic status (SES), sex, marital status, and educational level have received considerable attention. Usually, studies have measured physical activity with instruments such as accelerometers and questionnaires to evaluate their perception [16,17,18]. The scientific evidence has shown various results associated with this issue. 

Several studies focused on age and sex have indicated that older people performed lower physical activity than younger people, and men have demonstrated to be more active physically than women [19,20,21]. Nevertheless, the differences by sex differed from other results that showed that men and women reported similar physical activity levels [22]. The difference in physical activity levels between men and women may be due to cultural aspects since men are usually more motivated to participate in physical activities starting in adolescence [23]. In fact, in the Chilean context, it has been found that from the school stage, there are differences in physical activity levels between men and women and in their physical abilities [24]. The previous literature has highlighted a significant gap in the motivations for physical activity according to sex, as activities that require greater strength and competence tend to be more associated with men [25,26]. However, it is also possible that there are biological differences in the physiological response to physical activity, which could lead to variations in tolerance thresholds and physical capacity between men and women [27,28]. Although evidence has indicated a general tendency for differences in physical activity levels between men and women, physical activity is reduced and declines with age for both sexes [22]. With aging, there is usually a reduction in daily physical activity, which could be linked to the accumulation of deficiencies in various physiological functions essential to carrying out activities that require more vigorous movement [29]. However, it has also been observed that physical activity levels decrease over time from the school stage, which may be associated with the level of motivation for physical activity in school classes [30]. Motivation to engage in physical activity is critical, requiring educational, social, and family support to maintain it in young people, which has repercussions on healthy lifestyle habits into adulthood, with the environment also being a fundamental factor in promoting the development of physical activity [31]. Specifically, the sociocultural environment, which includes socioeconomic and educational aspects, also plays a fundamental role since it is documented that people with higher educational and socioeconomic status participate in more physical activities [32]. This situation could be because a socially and economically disadvantaged environment offers fewer opportunities for physical activity [33], as the conditions of the available spaces are usually less adequate.

Regarding the association between marital status and physical activity, some research findings have shown that married people spend less time on exercise than unmarried people [34]. In fact, according to Puciato and Rozpara [35], single people follow the WHO physical activity recommendations more closely, possibly because people who marry or cohabit with a partner begin to have other responsibilities such as housework and childcare, leaving less free time for exercise.

Sociodemographic factors may be less or more beneficial for physical activity. However, changes in the pandemic work mode due to restrictions during COVID-19 could be closely related to people’s level of physical activity, which is an interesting variable to consider in this issue. Working changes related to COVID-19 have been associated with a higher inactive physical lifestyle [36,37]. Research carried out during the pandemic lockdown has demonstrated that physical activity performed by people working at their job site was higher than those working remotely [8].

Considering the association between sociodemographic variables and their relationship with physical activity levels, it is essential to note that in the Chilean context, this association is similar to those of other countries. First, it has also been found that those with higher socioeconomic and educational levels tend to perform higher levels of physical activity [38]. Also, it has been found that women tend to engage in less vigorous and moderate physical activity than men, with a gap in these activities in work, leisure, and active transportation contexts [39]. Given the gender differences in physical activity levels in leisure and work contexts, it would be interesting to analyze whether these differences continue to exist in post-pandemic times since there is currently more significant teleworking. In addition, Chile is characterized as a Latin American country with a rapidly aging population, which has had significant consequences in the causes of death by COVID-19 in older people, especially those who have already suffered from specific pathologies [40]. Under this view, it would be relevant to investigate whether the COVID-19 pandemic continues to affect physical activity levels in different age groups since, according to the WHO, this situation continues to significantly impact the quality of life, especially in people with non-communicable diseases, with age being a risk factor [41]. Regarding the association between marital status and physical activity, it would be essential to know if, in the Chilean context, there is also a link between these two variables. This aspect would be interesting to analyze since it is known that in Chile, married older adults with a higher level of physical activity live longer than those who are married but are less physically active [42].

It should be noted that the results of previous studies that evaluate perceived physical activity, that is, through self-reports that collect information on the activity performed during a week, show levels of physical activity that are in line with the recommendations established by the WHO for the general population [43,44]. Several studies have collected information on perceived physical activity in Chileans, revealing its association with sociodemographic variables, which detail the intensity of activities and provide a comprehensive view of the contexts and types of physical activity in daily life [45,46,47].

Based on the evidence reviewed above, the overall aim of the current study is to examine the relationship between sociodemographic variables (i.e., sex, age, marital status, educational level, SES, and working mode) and physical activity levels (vigorous, moderate, and walking) declared by Chilean adults during the COVID-19 pandemic. Following the references mentioned in the introduction, we hypothesized a higher physical activity in men, people with higher education and SES, single and younger individuals, and those working at their job site. Therefore, we focused on the following research question: How are levels of physical activity reported by Chilean adults related to sociodemographic factors such as age, sex, marital status, socioeconomic and educational levels, and their working mode after the pandemic period?

## 2. Materials and Methods

### 2.1. Participants

A non-probabilistic sample of Chilean adult respondents was recruited. The following criteria were used to include the study participants: (a) reside in Chile at the moment of participation and (b) people aged 18 or over. This study adopted a quantitative approach with a correlational methodology and a descriptive cross-sectional design.

An online survey was promoted through emails and telephone calls to students and academics from universities where researchers worked from December 2022 to March 2023. Participants accessed a link to give their informed consent online. In total, 512 participants formed the original sample; however, 19 participants made a mistake when writing their date of birth, 6 did not answer their household income, 3 made a mistake when answering the IPAQ-SF, and 1 lived in another country. The study adopted a quantitative approach with a correlational methodology and a descriptive cross-sectional design. The final sample in the current study comprised 483 Chilean adults, 159 men (32.9%) and 324 women (67.1%) aged from 18 to 69 years old (36.5 ± 12.0). While 58% were not cohabiting with a partner or were unmarried, 42% were married or cohabiting with a partner. Overall, 32% of the respondents completed high school, 47% had a university or technical degree, and 21% had completed post-graduate studies. Almost all the participants (56.5%) worked online, 18.6% in hybrid mode, 10.4% at their job site, and 14.5% were not working. The household income was USD 72 to USD 12,000 (1883.2 ± 1686.7).

### 2.2. Measures 

An ad hoc sociodemographic online survey included questions about the characteristics of participants, such as sex, age, educational level, household income, marital status, working mode, and city and region where they were residing. This last variable was considered an exclusion criterion for people living in different countries.

The International Physical Activity Questionnaire-Short Form (IPAQ-SF) [48] was applied to estimate the physical activity level of subjects. The instrument has a version translated into Spanish [49] used in this study. The IPAQ-SF is a self-administered questionnaire that estimates physical activity in the last seven days. Recorded activities corresponding to vigorous, moderate, walking, and sitting time are converted to daily minutes. Using a physical activity calculation method given on the IPAQ website [50], it was possible to obtain the levels into which the subjects can be classified. Only the first three activities were considered for this study since energy expenditure was estimated. Therefore, sitting time was not considered.

### 2.3. Data Analysis

The data were analyzed in several stages. Firstly, the Kolmogorov–Smirnov test was used to verify the normality of the variables’ distribution, following the recommendation of Rivas-Ruiz et al. [51]. In all cases, the variables showed a normal distribution. Subsequently, sociodemographic factors were divided into categories. Specifically, sex was classified as women and men; the age in emerging adulthood (18–25 years old) according to Arnett criteria [52], young adulthood (26–45 years old), and older adulthood (46–69 years old), taking as a reference to Mesters et al. [53]. Marital status was categorized as single/unmarried and cohabiting with a partner/married. The educational level was divided into completed high school, university or technical degree, and post-graduate. Socioeconomic status was classified considering monthly household as low (USD 840 or less), middle (USD 841 to 2313), and high (USD 2314 or more), taking as reference the Chilean Association of Market Researchers 2019 criteria [54], and working mode as online, hybrid, a job site, and not working. 

The bivariate associations between sociodemographic factors and the IPAQ-SF scores were tested and quantified by parametric tests based on comparisons of means. Specifically, mean IPAQ-SF scores were compared via the Student *t*-test (for comparing two independent means) or robust ANOVA, followed by post hoc Bonferroni (for comparing more than two independent means). Effect sizes were computed using Cohen’s d and eta-squared (η^2^). All statistical analyses were carried out using SPSS version 27.0 for Windows.

## 3. Results

### Sociodemographic Factors and Physical Activity’s Levels Outcomes

The main results of the study are presented in Table 1. It shows that men declared significantly higher vigorous (t (252.54) = 2.27; *p* = 0.02; d = 0.24) and moderated (t (253.51) = 2.44; *p* = 0.02; d = 0.26) physical activity than women. A statistically significant effect of the people’s age on vigorous physical activity (F (2,480) = 6.69; *p* = 0.001; η^2^ = 0.03) was also found. In this regard, the group of people aged 18 to 25, emerging adulthood, showed significantly higher scores in vigorous physical activity than those aged 26 to 45 (young adulthood). 

Additionally, there was a significant association between the fact that people were not cohabiting with a partner or unmarried and scores corresponding to vigorous physical activity (t (477.70) = 3.5377; *p* ≤ 0.001; d = 0.36). Expressly, single people declared they performed significantly higher levels of vigorous activity. 

For working mode, people working at their job site showed higher mean scores in walking than people working in hybrid mode (F (2,480) = 4.26; *p* = 0.001; η^2^ = 0.02), online (F (2,480) = 4.26; *p* < 0.001; η^2^ = 0.02), and not working (F (2,480) = 4.26; *p* = 0.005; η^2^ = 0.02).

Respecting socioeconomic status, higher mean scores were found in vigorous physical activity in people with the lowest compared with the middle household income (F (2,480) = 4.26; *p* = 0.01; η^2^ = 0.02).

## 4. Discussion

This study aimed to examine the relationship between sociodemographic variables (i.e., sex, age, marital status, educational level, socioeconomic status, and working mode) and physical activity levels declared by Chilean adults in the period following the restrictions imposed due to the COVID-19 pandemic. Comparative analyses showed significant differences between vigorous, moderate, and walking activities declared by Chilean adults based on certain sociodemographic variables, especially in vigorous activities. Specifically, our results showed that men perceived that they performed more vigorous and moderated physical activity than women. There is solid evidence from studies of different cultures before the COVID-19 pandemic showing that men are usually more active than women [55,56,57], concordant with our results. Moreover, the evidenced difference found in this study could be emphasized by the evidence provided by a recent study in Chile showing that women have significantly decreased their physical activity during the COVID-19 pandemic lockdown [58]. Despite this result, it is crucial to consider that the difference between men and women could be possibly because men usually feel more intrinsically motivated to participate in activities requiring more vigorous movements than women [59]. In addition, as noted in the introduction, cultural and physiological causes may influence these differences [23,24,25,26,27,28].

Regarding the difference in physical activity between participants’ ages, previous studies found that people are more physically active when younger, but this activity decreases with age [60,61]. This situation might be explained by the possibility of a low level of physical activity being related to physiological changes in the musculoskeletal system as age progresses, imposing consequences such as reduced physical activity [62]. However, no significant differences were found with subjects in older adulthood (46–69 years old). In a study conducted by McCarthy et al. [63], older people were likelier to maintain or increase their physical activity levels during confinement than younger people who decreased it substantially. This was probably because younger people had a higher activity level before implementing the lockdown, which matched the older age groups who maintained it. After all, it was already low before the pandemic.

Despite this, our hypothesis that young people would report higher physical activity levels than older people was partially fulfilled. In particular, the emerging adulthood group, aged between 18 and 25, reported a higher level of vigorous activity than those in emerging adulthood (26–45 years old). These differences were probably found because life transitions during adulthood used to be associated with lower physical activities, including the beginning of the working world, responsibilities in the home, marriage, or parenthood [64]. These findings are particularly noteworthy as they indicate that the pandemic continues to impact younger individuals’ physical activity levels. According to a study by Katewongsa et al. [65], recovery to physical activity after the pandemic was faster in older people, whereas in younger people, this recovery was slower. In our findings, the decrease in physical activity has been so substantial that there are no significant differences when compared between young and older adults. However, among younger groups, variations due to sociodemographic factors, such as changing lifestyles, have emerged. Following Katewongsa’s study [65], it is highlighted that socioeconomic status may influence the slowness of recovery in physical activity. This suggests that the pandemic may have had a lasting effect on the physical activity levels of emerging and young adults, persisting into the present day.

Our results also showed that unmarried or non-cohabiting people declared more vigorous physical activity than married or cohabiting participants. Previous studies before the COVID-19 pandemic demonstrated similar results to ours [66,67]. A possible cause of this association could be the type of leisure-time physical activity in married people, linked to responsibilities and roles appropriately related to their identity [68]. Future research must consider the type of physical activity performed by married and unmarried people; variables not analyzed in this study could provide relevant information regarding the relationship of these variables with the level of physical activity declared as a consequence after a period of confinement in the pandemic.

Another aspect to consider in this research is the association between physical activity, SES, and educational level. Our results differed from what we hypothesized, and we did not find a significant difference between higher and lower levels of SES and education regarding physical activity. The previous literature has described that there are significant differences between physical activity levels according to socioeconomic and educational level, since people with fewer resources tend to have a lower educational level and, in turn, tend to have less healthy habits, including physical inactivity [69]; it is helpful to specify in which tasks people are more or less active according to their routine. For example, it has been found that middle-class people perform more intense physical activity in occupational activities, while people with fewer resources perform more household chores; and those with higher incomes perform more exercise, are more active when moving, but spend more time sitting [70]. It is essential to analyze, in the Chilean context, what activities they perform in their daily lives because it is well known that the pandemic led to subjects spending more time sitting, especially in the workplace [71], while people of lower socioeconomic level tend to work in unskilled jobs in which they need to perform face-to-face work. In fact, in Chile, it was found that 92% of unskilled workers performed face-to-face work, compared to only 29% of senior executives and entrepreneurs [72], probably bringing as a consequence that people who perform unskilled work are more mobile at work. 

Considering the preceding, our results showed that people working in the on-site mode walked more than those working in hybrid, online, and non-working modes. A systematic review that analyzed research from different countries concluded that confinement and working from home affected workers’ physical activity levels [73]. This could explain why, in our sample, those who maintained working at their job site walked significantly more than those who did not. This finding is interesting since it has been shown that commuting to work, which could include higher intensity activities, would affect workers who do not attend work at their job site [74]. Therefore, it is important to continue researching the association between work modalities, physical activity, and sedentary habits since the confinement that occurred due to the COVID-19 pandemic may have long-term repercussions on this issue.

Although our study does not directly compare physical activity levels before and after the COVID-19 pandemic, the existing literature indicates that both periods have seen notable similarities and differences in activity patterns among various demographic groups [63,75,76]. For example, men consistently report higher levels of vigorous physical activity than women, a trend observed both before and during the pandemic [19,20,21]. However, the pandemic appears to have exacerbated certain disparities, particularly among younger adults [65] and women, as in the Ramirez study et al. [77], where a decline in physical activity persists even as restrictions have eased. This suggests that, for some groups, the pandemic’s impact on physical activity levels has had lasting effects that continue to influence behavior to this day. The evidence supports the notion that while some sociodemographic factors may have been consistent, the pandemic introduced new challenges and shifts that have continued to affect physical activity levels in specific segments of the population, which can affect people’s health, especially weight gain, a problem that led to the COVID-19 pandemic and has had repercussions to date, according to the WHO [41].

## 5. Limitations and Future Directions for Research

The present research contributes to the state of the art on the importance of sociodemographic factors on the physical activity affected by the COVID-19 pandemic. However, the study presented several limitations when interpreting the results. First, cause–effect relationships cannot be determined because only associations between sociodemographic variables and IPAQ were calculated. Nevertheless, our results were supported by previous studies that have analyzed the relationships between sociodemographic variables and physical activity [6,8,58].

Other limitations include the instrument we used to measure sociodemographic factors and physical activity. On the one hand, an ad hoc sociodemographic questionnaire could be too specific and influenced by the biases and limitations of the current research design, which may result in a data collection that does not cover all the relevant aspects of the sociodemographic variables, thus limiting the comprehensive understanding of the study context. Despite this fact, many studies that measure physical activity levels in different age groups use sociodemographic questionnaires with characteristics similar to those of our research [20,78,79,80] since they tend to be the most common factors for assessing variables that may be related to physical activity, such as age, sex, educational level, and socioeconomic context, and that, as noted in the introduction, have been shown to have a significant relationship with movement behavior patterns [19,20,21,22,23,24,25,26,27,28,29,30,31,32,33,34,35,36,37]. On the other hand, the tool to measure physical activity was a self-report rather than an objective observation measurement tool, such as accelerometers and specific physical activity questionnaires at work, as applied in other studies [16,60,81]. However, the IPAQ-SF is a recommended and qualified instrument for investigating perceived physical activity because it is one of the most widely used self-report questionnaires in people between 15 and 69 years of age in different countries to assess physical activity [49].

One of the drawbacks that could affect the interpretation of the results of our study is that the effect sizes are small. Therefore, the strength of the relationship between the sociodemographic variables and the level of physical activity was low to medium [82], even though there were statistically significant differences when the groups of the population studied were compared. In this context, the relevance of the complement between reporting effect sizes and statistical significance is that the findings are not due to chance [83], which was probably the case in this study.

Moreover, studies of this nature in which the relationship between the self-reported physical activity level and sociodemographic variables has been analyzed tend to be conducted with larger samples than our study [17,84,85]. However, in research that has been conducted after the pandemic, as in our study, the number of participants is usually equal to or less than the number of participants in this study [86,87,88], suggesting that our sample size may be adequate to obtain reliable results within the context of the study, allowing us to capture relevant trends.

However, we used convenience sampling, selecting participants based on accessibility. While this approach was practical, it limits the generalizability of our results to the specific sample studied. The relationships between sociodemographic variables and physical activity levels observed here may not extend to the broader population. Additionally, the use of convenience sampling can lead to variability in effect sizes and may contribute to inconsistent findings in developmental science [89]. Therefore, our findings should be interpreted with caution.

Despite the above limitations, the results of our study provide valuable information that can be used by institutions, physical educators, and coaches to develop interventions aimed at increasing physical activity levels in various demographic groups. For example, the finding that men and younger adults engage in more vigorous physical activity suggests the need for programs encouraging similar activity levels among women and older adults. Agencies such as the National Service for the Elderly (SENAMA) [90] can use these data to continue to design initiatives that promote physical activity in older adults, while the Ministry of Women [91] could continue to develop activities that contribute to policies that address the specific barriers women face in participating in more vigorous physical activity. In addition, the Program Choose to Live Health [92] may continue implementing strategies that ensure equitable access to physical activity opportunities, especially for those in lower socioeconomic sectors, where greater physical activity was observed, possibly due to manual labor or economic necessity. Physical educators and trainers can use this knowledge to design gender-sensitive and age-appropriate exercise routines that respond to their client’s specific needs and preferences. In addition to recognizing the influence of marital status on physical activity, unmarried individuals reported higher levels of vigorous activity, encouraging greater participation through family physical activity strategies. Understanding these sociocultural variables allows for a more nuanced approach to promoting physical activity, ensuring effective and culturally sensitive interventions.

Our findings consider sociodemographic variables as a whole, providing essential data that may contribute to future research that analyzes the physical activity levels among groups considering specific sociodemographic variables in more detail. In that sense, exploring how the interaction of sociodemographic factors influences physical activity and how measures taken during the pandemic may have affected physical activity levels to the present day is recommended. Such an approach would allow a deeper understanding of the dynamics affecting physical activity in different groups considering marital status [35], sex, age [16,19,20,21], socioeconomic level [33,70], and work mode [8], which have examined these variables individually.

## 6. Conclusions

Sociodemographic factors continue to influence physical activity, as before the COVID-19 pandemic. However, the public health measures that were implemented had a negative impact on motor behaviors of higher intensity that, apparently, have been slow to recover, even after terminating the confinement measures. The most common sociodemographic variables related to physical activity were sex, age, marital status, and SES. Nevertheless, the absence of on-site work may affect walking intensity or the ability to engage in strenuous activity during work, which is a worrying situation because many people have maintained this work modality. This is a critical issue in studies related to physical activity in the context of the COVID-19 post pandemic. Therefore, our results strongly advocate promoting strategies that increase physical activity to avert potential health problems that the pandemic brought about and have had repercussions up to the present day. The findings of our study underscore the importance of designing tailored interventions to promote physical activity in different demographic groups, taking into account factors such as sex, age, and marital status. Institutions can use this information to develop more inclusive and equitable programs that address the specific barriers certain groups face, such as women and older adults, in their participation in physical activities that generate a greater likelihood of success and continuity. Likewise, physical educators and coaches should adapt their approaches to create routines that consider the needs and motivations of each group, promoting a more inclusive and culturally sensitive environment.

## Figures and Tables

**Table 1:** Descriptive summary of IPAQ-SF scores by sociodemographic factors.

	Vigorous	Moderate	Walking
	M ± SD (Min–Max)	M ± SD (Min–Max)	M ± SD (Min–Max)
Sex			
Men (*n* = 159)	1161.51 ± 1772.48 (0−10,080)	569.76 ± 989.89 (0−5040)	524.78 ± 694.34 (0−4158)
Women (*n* = 324)	799.63 ± 1362.55 (0−864)	352.31 ± 764.91 (0−5040)	503.50 ± 931.94 (0−4158)
Cohen’s d	0.24 *	0.26 *	0.03
Adulthood stage categories			
Emerging (*n* = 100)	1376 ± 1726.12 (0−8640)	487.80 ± 815.85 (0−4200)	453.42 ± 590.84 (0−2772)
Young (*n* = 283)	740.78 ± 1302.10 (0−7200)	372.23 ± 823.72 ** (0−5040)	488.88 ± 922.38 (0−4158)
Older (*n* = 100)	965.20 ± 1760.50 (0−10,080)	506.20 ± 952.24 (0−5040)	628.82 ± 901.22 (0−4158)
η^2^	0.03 **	0.01	0.01
Marital status			
Single (*n* = 280)	1116.43 ± 1637.52 (0−10,080)	478.00 ± 886.52 (0−5040)	499.18 ± 786.81 (0−4158)
Cohabiting/married (*n* = 203)	646.11 ± 1289.43 (0−7200)	349.26 ± 794.88 (0−5040)	526.13 ± 954.26 (0−4158)
Cohen’s d	0.31 **	0.15	0.03
Educational level			
Completed high school (*n* = 157)	1747.77 ± 1668.93 (0−8640)	528.28 ± 882.49 (0−5040)	579.60 ± 849.27 (0−4158)
Graduated (*n* = 225)	821.87 ± 1375.97 (0−7200)	348.27 ± 762.47 (0−5040)	499.62 ± 887.88 (0−4158)
Postgraduated (*n* = 101)	778.61 ± 1546.21 (0−1080)	430.10 ± 970.21 (0−5040)	427.37 ± 813.45 (0−4158)
η^2^	0.01	0.01	0.00
Working mode			
Without modality (*n* = 70)	849.14 ± 1346.83 (0−8640)	336.29 ± 616.05 (0−3360)	525.17 ± 849.94 (0−4158)
Online (*n* = 273)	934.95 ± 1449.85 (0−8400)	379.41 ± 756.84 (0−5040)	412.80 ± 738.63 (0−4158)
Hybrid (*n* = 90)	777.33 ± 1495.46 (0−7200)	477.33 ± 1034.33 (0−5040)	497.93 ± 796.44 (0−4158)
On site (*n* = 50)	1182.40 ± 2062.12 (0−1080)	693.20 ± 1166.57 (0−5040)	1046.10 ± 1317.64 (0−4158)
η^2^	0.01	0.01	0.05 **
Socioeconomic status			
Low (*n* = 131)	1210.99 ± 1646.52 (0−8640)	546.41 ± 934.79 (0−5040)	590.60 ± 929.60 (0−4158)
Middle (*n* = 221)	727.96 ± 1358.00 (0−1080)	381.23 ± 849.75 (0−5040)	518.22 ± 912.19 (0−4158)
High (*n* = 131)	948.40 ± 1604.45 (0−8400)	373.34 ± 752.90 (0−5040)	417.41 ± 675.73 (0−4158)
η^2^	0.02 *	0.01	0.01

* *p* ≤ 0.01; ** *p* ≤ 0.001; M: mean; SD: standard deviation; Min: Minimal value; Max: Maximal value.

## Data Availability

The data supporting this study can be requested from the corresponding author. The data are not publicly accessible because they contain sensitive information. An anonymized version of the database, including the relevant variables for reproducing the analyses, has been created and is available upon request.
